# Stable High-Efficiency Two-Dimensional Perovskite Solar Cells Via Bromine Incorporation

**DOI:** 10.1186/s11671-020-03406-w

**Published:** 2020-10-01

**Authors:** Feng Han, Wenyao Yang, Hao Li, Lei Zhu

**Affiliations:** 1 Xi’an Technological University, Xi’an, 710021 People’s Republic of China; 2 Chongqing Engineering Research Center of New Energy Storage Devices and Applications, Chongqing University of Arts and Sciences, Chongqing, 402160 People’s Republic of China; 3 State Key Laboratory of Electronic Thin Films and Integrated Devices, and School of Optoelectronic Science and Engineering, University of Electronic Science and Technology of China (UESTC), Chengdu, 610054 Sichuan People’s Republic of China; 4 Xi’an Institute of Applied Optics, Xi’an, 710100 People’s Republic of China

**Keywords:** Two-dimensional perovskite, Bromine incorporation, Stability

## Abstract

Two-dimensional (2D) organic-inorganic perovskites as one of the most important photovoltaic material used in solar cells have attracted remarkable attention. These 2D perovskites exhibit superior environmental stability and wide tunability of their optoelectronic properties. However, their photovoltaic performance is far behind those of traditional three-dimensional (3D) perovskites. In this work, we demonstrate the power conversion efficiency (*PCE*) of 2D perovskite solar cells (PVSCs) is greatly improved from 3.01% for initial to 12.19% by the incorporation of PbBr_2_. The enhanced efficiency is attributed to superior surface quality, enhanced crystallinity, and the resulting reduced trap-state density. Furthermore, PbBr_2_ incorporated devices without encapsulation show excellent humidity stability, illumination stability, and thermal stability. This work provides a universal and viable avenue toward efficient and stable 2D PVSCs.

## Introduction

During the past decade, the hybrid organic-inorganic perovskites have drawn significant attention as promising photo-voltage materials due to their easy preparation process and excellent optoelectronic characteristics, such as small exciton binding energy, appropriate bandgap, great light absorption, and long exciton diffusion length [1–6]. At present, the highest certified *PCE* has exceeded 25% of 3D PVSCs [7]. Unfortunately, the stability issue of 3D perovskite hinders the commercial application of perovskite solar cells. For example, CH_3_NH_3_PbI_3_ (MAPbI_3_) perovskite will degrade rapidly when exposed to light for long periods of time or exposed to moisture [8, 9]. This problem prompted researchers to work hard to improve the stability of perovskite materials.

Recently, 2D perovskite (RNH_3_)_2_A_*n*−1_M_*n*_X_3*n*+1_ (Ruddlesden-Popper phase) have been developed due to their outstanding moisture resistance, wherein R is a long-chain organic group or bulky organic group, A stands for small organic cation (MA^+^, FA^+^, or Cs^+^), M corresponds to the B-cation in the three-dimensional perovskite (i.e., Pb^2+^ and Sn^2+^ ), X is halide anion (I^−^, Br^−^ and Cl^−^), and *n* is the number of octahedrons in each individual perovskite layer which defined the number of 2D perovskite [10–17]. Owing to the stronger van der Waals interaction between the blocked organic molecules and the [MX_6_]^4−^ unit, 2D perovskite exhibits better stability than 3D perovskite [10]. However, the large exciton binding energy of 2D perovskite makes exciton dissociation more difficult [18]. Meanwhile, the insulation of the organic spacer layer hinders the transport of carriers, resulting in a reduction in photo-generated current [12]. Therefore, the PCE of 2D PVSCs lags far behind that of their 3D counterparts.

Different methods have been implemented to improve the performance of 2D PVSCs, including additive engineering [19–24], component regulation [25–33], interfacial engineering [34–37], and preparation process [38–40]. Halogen ions show great potential to improve the performance of the device in 3D PVSCs. For example, a small amount of chloride in 3D perovskite can extend the crystal crystallization time, change the crystal growth direction, reduce the density of trap states, and increase the diffusion length of photo-generated carriers [41–44]. Meanwhile, previous work proves that a small amount of bromine doped 3D perovskite enhances the stability, suppresses ion migration, and reduces trap-state density [45]. Considering the composition of 2D perovskite, it is necessary to carry out research on halogen regulation. However, only limited work has been carried on the influence of 2D perovskite halogen regulation on device performance. Liu and his co-worker have found that chloride plays a critical role to improve perovskite morphology. By regulating the chloride ratio of the precursor solution, the 2D perovskite film with increased grain size, enhanced crystallinity, and uniform surface was obtained. As results, the PCE of 2D PVSCs with excellent stability was remarkably improved from 6.52 to 12.78% [46]. These results confirm that halogen regulation can improve the performance of 2D PVSCs.

In this work, we investigated the influence of bromine on the opto-electronic properties of the 2D perovskite by using n-butylamine (BA) spacer. Bromine was incorporated by using lead (II) bromide (PbBr_2_). It is demonstrated that the incorporation of an appropriate amount of bromine is able to facilitate the formation of high-quality 2D perovskite film, which results in the reduced defect states of 2D perovskite film and enhanced photovoltaic performance of 2D PVSCs. The PCE of 2D PVSCs is boosted from 3.66 to 12.4%. More interestingly, the optimal 2D PSVCs devices exhibit a significant improvement in humidity, illumination, and thermal stabilities.

## Method

### Materials and Solution Preparation

Lead (II) iodide (PbI_2_), PbBr_2_, n-butylammonium iodide (BAI), methylamine iodide (CH_3_NH_3_I, MAI), PEDOT:PSS (4083) aqueous solution, phenyl-C61-butyric acid methyl ester (PC_61_BM), and bathocuproine (BCP) were purchased from Xi’an Polymer Light Technology Cory. N,N-dimethylformamide (DMF), dimethyl sulfoxide (DMSO), and chlorobenzene were ordered from Sigma-Aldrich. Isopropanol was purchased from You Xuan Trade Co., Ltd. All reagents and solvents were used as received. The 2D perovskite BA_2_MA_4_Pb_5_I_16-10*x*_Br_10*x*_ (*n* = 5, *x =* 0, 5, 10, or 15%) precursor solution (0.8 M) was fabricated by adding BAI, MAI, PbI_2_, and PbBr_2_ with a molar ratio of 0.4:0.8:1-*x*:*x* in the mixed solvent of DMSO and DMF in 1:15 volume ratio.

### Device Fabrication

The indium tin oxide (ITO) substrates were cleaned by sequential sonication in detergent, acetone, absolute ethyl alcohol, and deionized water for 15 min each. The ITO substrates were dried in N_2_ flow and cleaned by UV–O_3_ treatment for 15 min. PEDOT:PSS aqueous solution was then spin-coated onto the ITO substrates under 5000 rpm for 30 s, followed by annealing at 150 °C for 15 min in air. Subsequently, the PEDOT:PSS/ITO substrates were transferred into a nitrogen glove box. The 2D perovskite solutions with different bromine content were spin-coated onto the preheated PEDOT:PSS/ITO substrates by a spin-coating process at 5000 rpm for 20 s and then annealing at 100 °C for 10 min. After annealing, the prepared PCBM solution (20 mg/mL in chlorobenzene) and BCP solution (0.5 mg/mL in isopropanol) were posited above on 2D perovskite film at 2000 rpm for 30 s and 5000 rpm for 30 s, respectively. Finally, thermally evaporation was implemented to prepare the electrodes Ag with thickness of 70 nm.

### Measurement and Characterization

The scanning electron microscope (FEI-Inspect F50, Holland), atomic force microscopy (Cypher S), and X-ray diffraction (Bruker D8 ADVANCE A25X) measurements were conducted based on the structure of ITO-etched glass/PEDOT:PSS/2D perovskite. The UV-visible absorption spectrum of 2D perovskite films on glasses was measured by Shimadzu 1500 spectrophotometer. PL spectrum was collected by Fluo Time 300 (Pico Quant) spectrofluorometer. The current density-voltage (*J-V*) characteristics of 2D PVSCs were collected using a Keithley 2400 Sourcemeter under AM 1.5G sun intensity irradiated by a Newport Corp solar simulator. The active area of the device is 0.04 cm^2^. The *J*-*V* curves were measured in the reverse (from 1.2 to 0 V) and forward (from 0 to 1.2 V) directions with a scanning rate of 0.23 V/s, fixed voltage interval of 0.0174 mV, and dwelling time of 10 ms. Dark current-voltage curves were measured in the same way under the dark condition.

## Results and Discussion

**Fig. 1:**
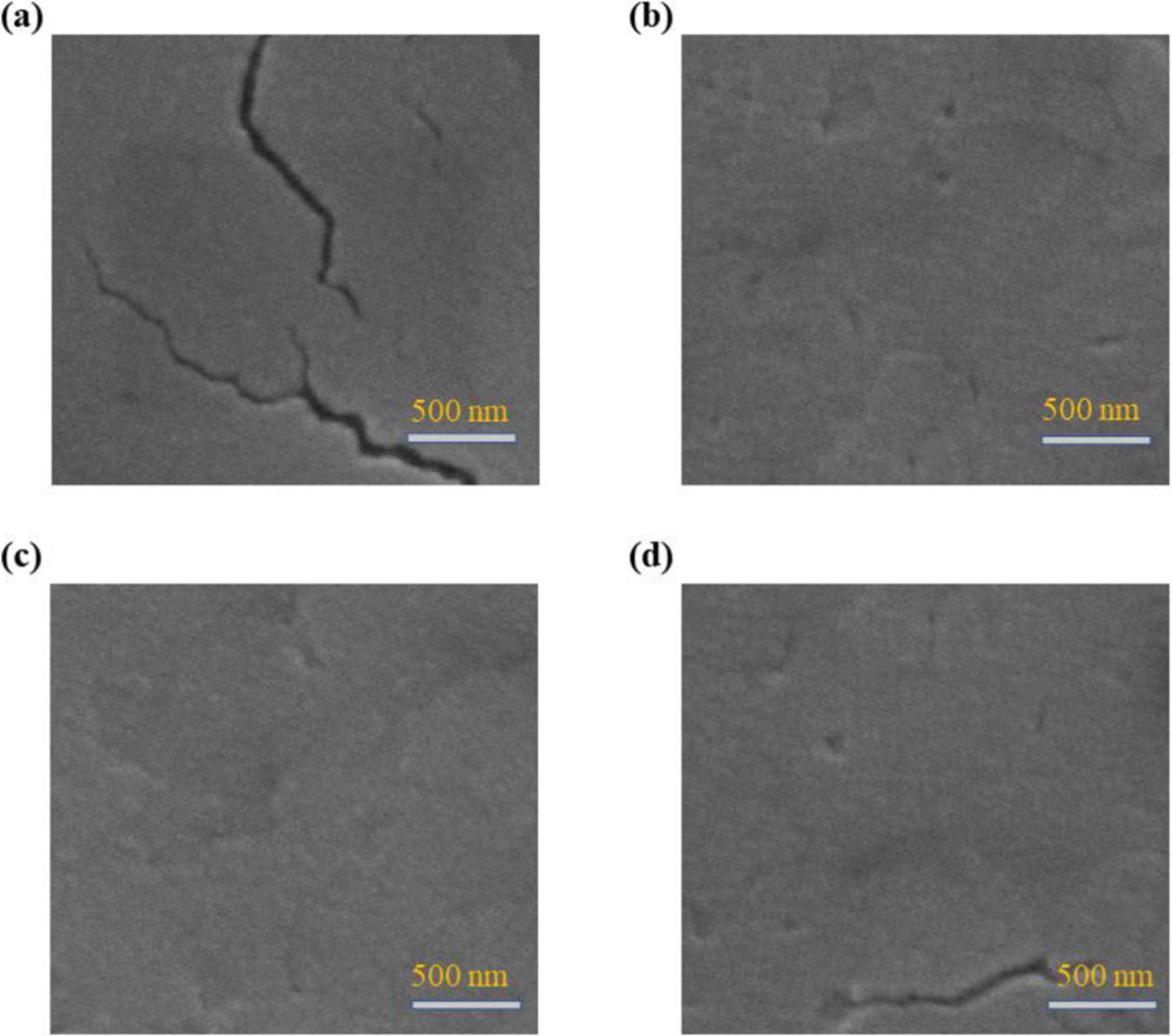
SEM images of BA_2_MA_4_Pb_5_I_16-10*x*_Br_10*x*_ films based on **a** 0% PbBr_2_, **b** 5% PbBr_2_, **c** 10% PbBr_2_, and **d** 15% PbBr_2_

**Fig. 2:**
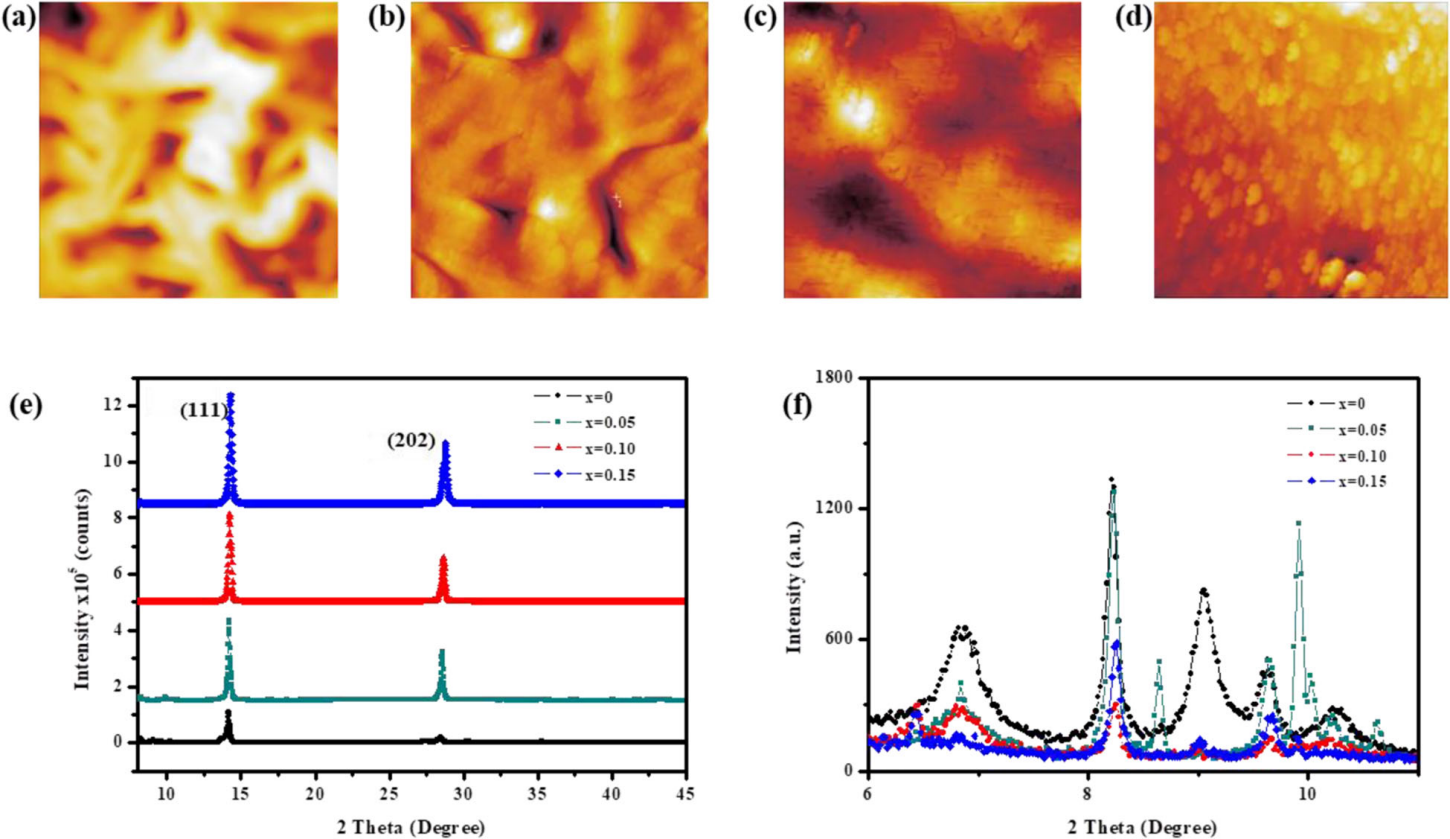
AFM images of BA_2_MA_4_Pb_5_I_16-10*x*_Br_10*x*_ films based on **a** 0% PbBr_2_, **b** 5% PbBr_2_, **c** 10% PbBr_2_, and **d** 15% PbBr_2_. X-ray diffraction patterns (**e**) and corresponding local enlarged image (**f**) of BA_2_MA_4_Pb_5_I_16-10*x*_Br_10*x*_ films with various amounts of PbBr_2_

The 2D perovskite films incorporated different amounts of bromine were prepared by a previously reported hot-casting method. By using this method, substrates are preheated to favor crystallization and orientation [40]. To investigate the effects of different amounts of PbBr_2_ in the 2D perovskite precursor solutions on the morphology of resultant film, a scanning electron microscope (SEM) and atomic force microscopy (AFM) measurements were carried out. As shown in Fig. 1a, the 2D perovskite BA_2_MA_4_Pb_5_I_16-10*x*_Br_10*x*_ film without bromine incorporation (*x* = 0%, denoted as control perovskite) exhibits a poor morphology with big cracks, indicating the low coverage and inferior compactness. The cracks are disappeared in the 2D perovskite film with 5 mol% PbBr_2_ content (*x* = 5%, denoted as perovskite-5%). However, the perovskite-5% film still shows some pinholes (Fig. 1b). In the case of the 2D perovskite film with 10 mol% PbBr_2_ content (*x* = 10%, denoted as perovskite-10%), the film surface becomes uniform and compact without any cracks or pinholes (Fig. 1c). As the PbBr_2_ content is further increased to 15 mol% (*x* = 15%, denoted as perovskite-15%), cracks appeared in the film again (Fig. 1d). The AFM images of 2D perovskite film with various amounts of PbBr_2_ are shown in Fig. 2a–d, which are consistent with the SEM results. The control perovskite film shows a rough surface with a high root-mean-squared roughness (RMS) value of 51.2 nm. The partial replacement of iodine with bromine greatly reduces the RMS value to 21.3 nm for perovskite-5% and 23.1 nm for perovskite-15%, respectively. Especially, the perovskite-10% film exhibits a quite smooth surface with the lowest RMS value of 10.7 nm due to the disappearance of cracks and pinholes. The above results indicate that incorporating an appropriate amount of bromine is beneficial to improve the uniformity and surface coverage of the 2D perovskite film. It is well known that cracks and pinholes in the film can lead to strong energetic disorder, cause recombination, impede charge transport, and weaken photovoltaic performance [47]. Therefore, obtaining a uniform and well-covered perovskite film is essential to improve device efficiency.

To investigate the impact of bromine on the crystal phase and crystallinity of 2D perovskite films, X-ray diffraction (XRD) measurements were performed. As shown in Fig. 2e, all films show two distinctive diffraction peaks at around 14.5° and 28.4°, which can be assigned to (111) and (202) crystallographic planes, respectively. Previous studies have suggested that both the (111) and (202) orientation allow the [(MA)_*n*−1_Pb_*n*_I_3*n*+1_]^2−^ slabs grow in vertical alignment to the PEDOT:PSS/ITO substrate [13, 23, 24]. Therefore, limited replacement of iodine with bromine is conducive to the formation of vertically oriented 2D perovskite film, as evidenced by the preferred intensity increase in the (111) and (202) peaks [48]. The vertical oriented 2D perovskite film allows more efficient transport of photon-induced carriers, improving photovoltaic performance of PVSC [23, 24]. On the one hand, the diffraction peaks at around 14.5° and 28.4° both become stronger upon the incorporation of bromine, suggesting the enhanced crystallinity of the perovskite film. On the other hand, the two peaks are gradually shifted towards higher angles upon the bromine incorporation, which is due to the smaller size of the bromine ion with respect to the iodine ion that shrinks the crystal lattice [13]. These gradual shifts in diffraction peak position prove that mixed BA_2_MA_4_Pb_5_I_16-10*x*_Br_10*x*_ perovskites are formed with bromine ion inserted in the crystal lattice. It is worth noting that all of the films show the peaks of (0 *k*0) reflections at low angles (< 10°), indicating the formation of 2D RP perovskite structures (Fig. 2f). However, the control film exhibits some diffraction peaks that could not be assigned to any typical 2D perovskite characteristic peak. The intensity of these undesired peaks is weakened upon the incorporation of bromine, giving rise to the lowest intensity in perovskite-10% film. This phenomenon suggests that the incorporation of moderate bromine can inhibit the formation of the impurity phases in the 2D perovskite film.

**Fig. 3:**
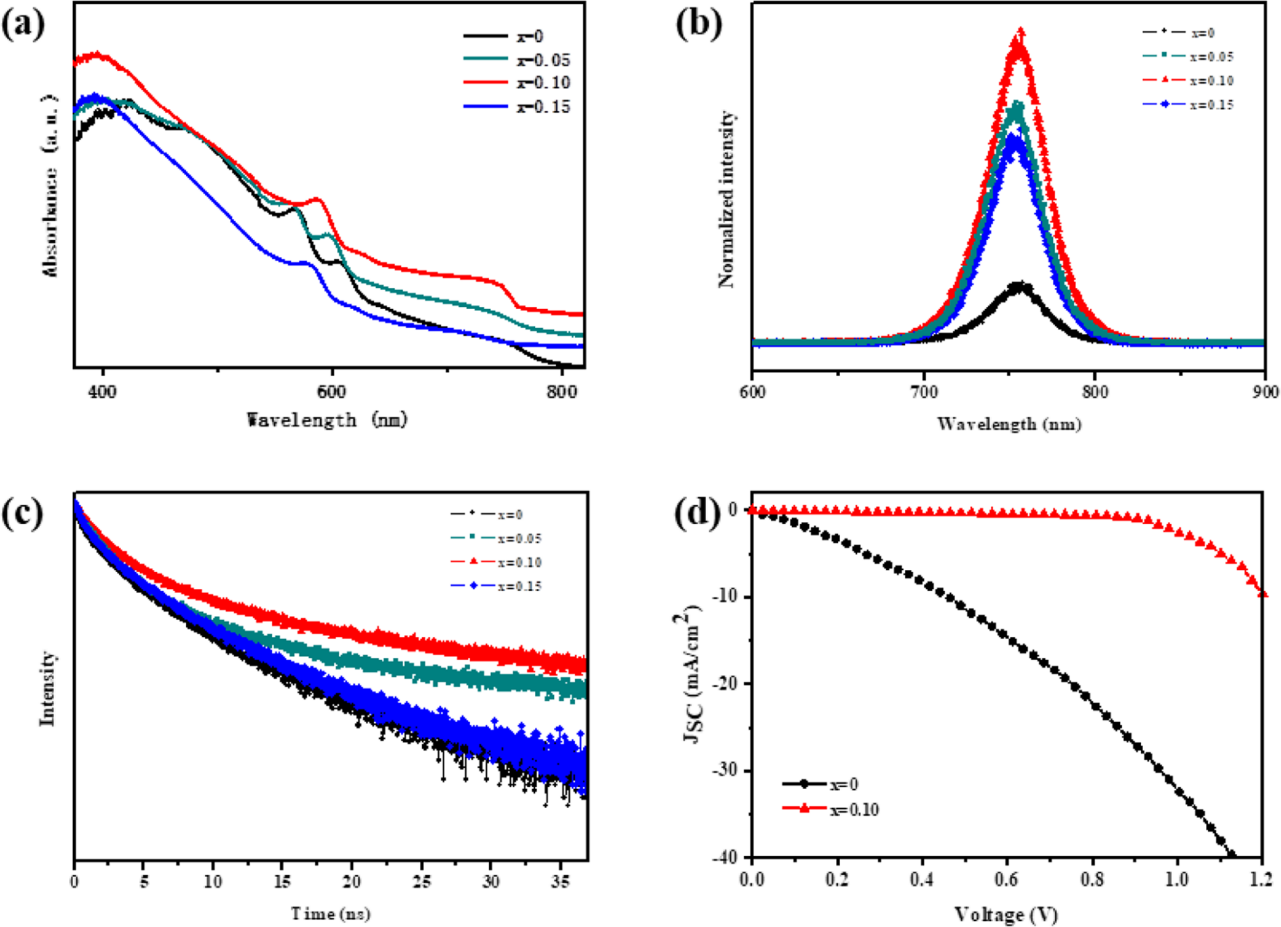
**a** Absorption spectra, **b** steady-state PL spectra, and **c** time-resolved PL curves of BA_2_MA_4_Pb_5_I_16-10*x*_Br_10*x*_ film with various amounts of PbBr_2_ spin-coated on glass substrates. **d** Dark current-voltage measurements of PVSCs based on the BA_2_MA_4_Pb_5_I_16-10*x*_Br_10*x*_ film with various amounts of PbBr_2_

Furthermore, the absorbance and photoluminescence (PL) measurements were carried out to understand the influence of bromine incorporation on the film optical properties, as summarized in Fig. 3a–c. Figure 3a shows the UV-visible absorption spectra of the 2D perovskite film with various amounts of PbBr_2_. All these films show distinctive exciton absorption peaks in the absorption spectra, which are assigned to 2D phases with *n* = 2, 3, and 4, although nominally prepared as “*n* = 5.” The perovskite-10% exhibits the enhanced absorbance intensity, resulting from a dense and uniform nature of the resultant film, as evidenced by the SEM and AFM images. Besides, the absorption edge of BA_2_MA_4_Pb_5_I_16-10*x*_Br_10*x*_ has a blue shift with the increase of *x* value, which proves the widening of bandgap [49]. Figure 3b presents the steady-state PL spectra of the 2D perovskite films deposited on glass substrates. As compared to the control sample showing the weakest PL signal, either perovskite-15% sample or perovskite-5% sample exhibits the increased PL signal, while the perovskite-10% sample shows the strongest PL signal. Remarkable PL enhancement is observed after incorporating bromine, indicating the reduced trap-state density in the PbBr_2_ treated films. Figure 3c displays the time-resolved PL decay spectra of the BA_2_MA_4_Pb_5_I_16-10*x*_Br_10*x*_ films spin-coated on glass substrates, which also proves the reduction of trap-state density in perovskite with the incorporation of bromine. The time-resolved PL curves were fitted with a two-exponential equation (Eq. (1)) containing a fast decay and a slow decay process, and the fitting parameters are summarized in Table 1. The fast decay (*τ*_*1*_) is considered to be the result of the quenching of carriers transport in the perovskite domain, and the slow decay (*τ*_*2*_) is the result of radiative recombination [50]. The average lifetime (*τ*) of 2D perovskite films are calculated according to Eq. (2). The perovskite-10% film presents the longest *τ* of 3.47 ns as compared to other films (i.e., 0.9 ns, 2.72 ns, and 1.31 ns for control film, perovskite-5% film, and perovskite-15% film, respectively), suggesting a slower recombination process with less defects.

1 $$ I(t)={\mathrm{A}}_1\exp \left(-\frac{t}{\tau_1}\right)+{\mathrm{A}}_2\exp \left(-\frac{t}{\tau_2}\right) $$

2 $$ \tau ={A}_1\times {\tau}_1+{A}_2\times {\tau}_2 $$

**Table 1:** Time-resolved PL parameters of BA_2_MA_4_Pb_5_I_16-10*x*_Br_10*x*_ films spin-coated on glass substrates

Sample	*A*_1_	*τ*_*1*_ (ns)	*A*_2_	*τ*_*2*_ (ns)	*τ* (ns)
0 (*x* = 0)	0.86	0.49	0.14	3.45	0.90
5 (*x* = 0.05)	0.80	1.32	0.20	6.08	2.72
10 (*x* = 0.10)	0.75	1.59	0.25	9.12	3.47
15 (*x* = 0.15)	0.83	0.76	0.17	4.04	1.31

In addition, to investigate whether reduced defect states arise from the PbBr_2_ when the 2D perovskite films are assembled in a PVSC structure, dark current-voltage curves of the corresponding devices were also collected (Fig. 3d). The dark current of the device based on the perovskite-10% film is much lower than that of the device based on the control film at the same voltage. The lower dark current of the device based on the perovskite-10% film indicates that the reduced defect states are indeed contributed by the bromine incorporation.

**Fig. 4:**
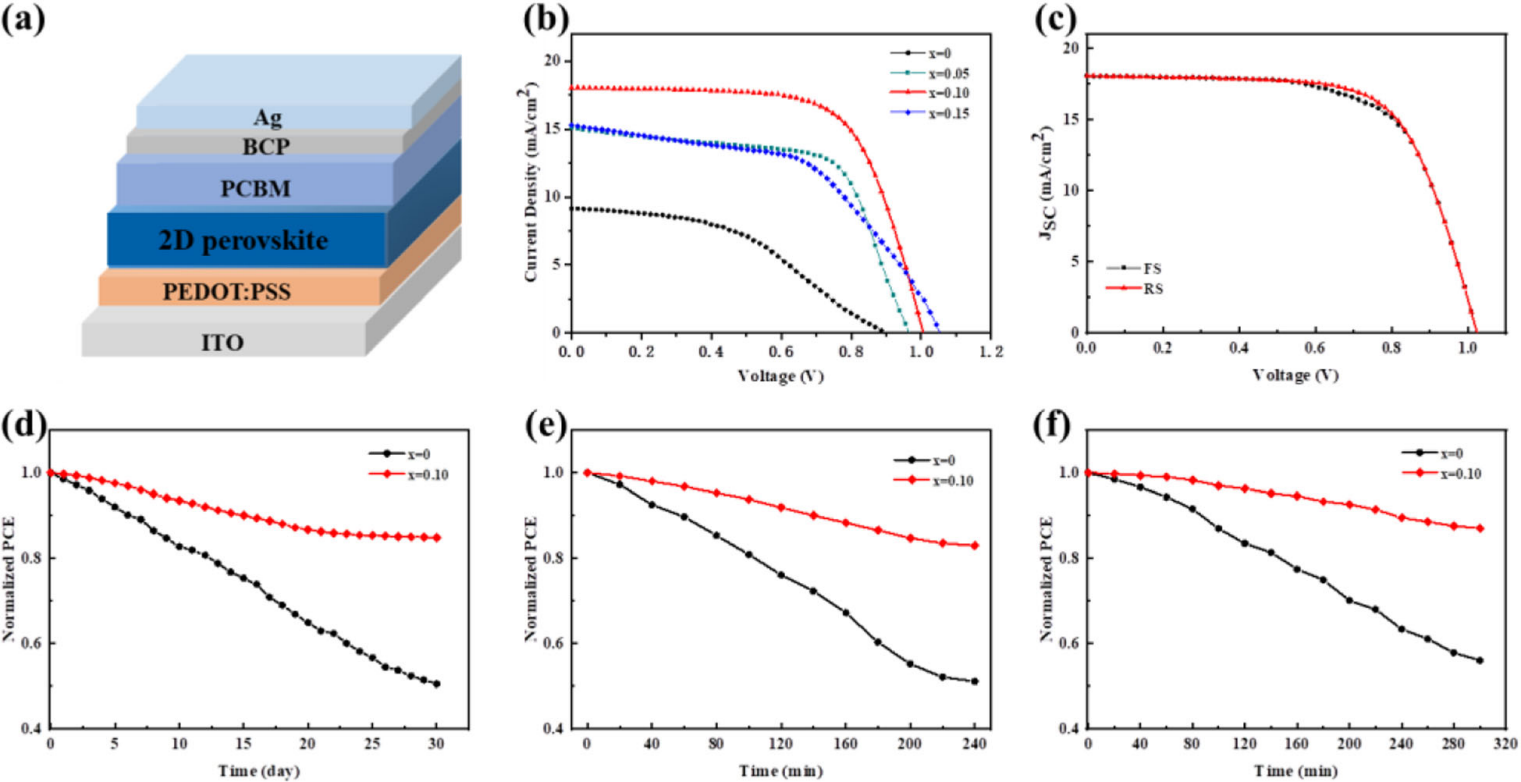
**a** The device architecture of PVSC. **b** *J-V* curves of PVSCs based on BA_2_MA_4_Pb_5_I_16-10*x*_Br_10*x*_ films with various amounts of PbBr_2_. **c** *J-V* curves of the best-performing device at different scan directions. **d** Humidity stability, **e** illumination stability, and **f** thermal stability of the unsealed device without and with 10 mol% PbBr_2_

It is shown PbBr_2_ in 2D perovskite films induced improved morphology, crystallinity, and opto-electronic properties. We fabricated PVSC devices with the planar p-i-n architecture as indium tin oxide (ITO)/PEDOT:PSS/BA_2_MA_4_Pb_5_I_16-10*x*_Br_10*x*_/PCBM/BCP/Ag. The *J-V* curves and the related parameters of the best-performing devices are shown in Fig. 4a and Table 2. The PVSCs based on the control perovskite film yielded a poor device performance, showing a champion *PCE* of 3.01% with an open-circuit voltage (*V*_oc_) of 0.89 V, a short-circuit current density (*J*_sc_) of 8.28 mA/cm^2^, and a fill factor (*FF*) of 40.79%. The introduction of bromine into the perovskite precursor remarkably increases the *PCE* of the device (Fig. 4a). The highest *PCE* of 12.19% with a *V*_oc_ of 1.02 V, a *J*_sc_ of 17.86 mA/cm^2^, and a fill factor (*FF*) of 66.91% was obtained in the 10 mol% PbBr_2_-treated device compared to 8.88% in the 5 mol% PbBr_2_-contained device and 7.85% in the 15 mol% PbBr_2_-contained device. In order to more accurately compare the performance of these devices, 20 devices for each case were fabricated. From statistical data (Fig. S1, Supporting Information), the device with 10 mol% bromine shows the relatively higher *V*_oc_ and *FF*, which is ascribed to the reduced trap-state density resulting from high-quality perovskite film, as discussed in Fig. 3b–d. The higher *V*_oc_ in Br-contained devices can also attribute to the increased bandgap. The bandgap of the BA_2_MA_4_Pb_5_I_16-10*x*_Br_10*x*_ increases with the increasing PbBr_2_ ratio, as evidenced by Fig. 3a [49]. Thus, the 15 mol% PbBr_2_-contained device shows the highest *V*_oc_. Moreover, the high *J*_sc_ in 10 mol% PbBr_2_-contained device can attribute to the increased light absorption and the efficient charge transport, as discussed above. The hysteresis of the devices based on the control perovskite film and perovskite-10% film was investigated by scanning the *J-V* curves in different directions (Fig. 4c and Fig. S2). The device based on the perovskite-10% exhibits slight hysteresis while serious hysteresis characteristic was observed in the device based on the control perovskite, indicating again the significant reduced defect states in the former case.

**Table 2:** Photovoltaic parameters of the champion devices based on BA_2_MA_4_Pb_5_I_16-10*x*_Br_10*x*_ films with various amounts of bromine

Sample	*V*_OC_ (V)	*J*_SC_ (mA/cm^2^)	*FF* (%)	*PCE* (%)
*x* = 0	0.89	8.28	40.79	3.01
*x* = 0.05	0.95	14.84	63.02	8.88
*x* = 0.10	1.02	17.86	66.91	12.19
*x* = 0.15	1.06	14.59	50.73	7.85

Furthermore, the incorporation of PbBr_2_ can effectively enhance the humidity, illumination, and thermal stability of the 2D PVSCs. The unsealed control device and device based on perovskite-10% were exposed to a relative humidity level of 45–60% at 25 °C for the humidity stability testing. The *PCE* of the control device declines to 50% of its original value within 30 days while the device based on perovskite-10% still maintains 85% of its initial efficiencies under identical conditions (Fig. 4d). Interestingly, the introduction of PbBr_2_ also enhances the illumination stability of the PVSCs. After being irradiated continuously under AM 1.5G sun intensity for 240 min, the devices retain more than 80% of the original *PCE* for perovskite-10% while only less than 50% for the control perovskite (Fig. 4e). The enhancement of thermal stability also confirmed by measurement. Both the control device and perovskite-10% device were thermally annealed at 85 °C in nitrogen atmosphere without encapsulation. As shown in Fig. 4f, the perovskite-10% device retains 83% of its initial *PCE* after 300 min, which is much higher than that of the control device (54%).

## Conclusion

In conclusion, we demonstrated that incorporating suitable bromine in precursor solution can improve the morphology of 2D perovskite films with enhanced crystallinity, leading to an improvement in opto-electronic properties in terms of absorbance and trap density. The outstanding film quality and opto-electronic properties yield an obvious enhancement in *PCE* from 3.01 to 12.19%. Moreover, the bromine incorporation enhances the tolerance of PVSCs to humidity, illumination, and thermal stability. These results prove that incorporating bromine is crucial to achieving stable high-performance 2D PVSCs.

## Additional File

**Additional file 1: Fig. S1.** Statistic distribution for (a) *V*_*oc*_, (b) *J*_*sc*_, (c) *FF*, and (d) *PCE* of 2D PVSCs based on BA_2_MA_4_Pb_5_I_16-10*x*_Br_10*x*_ films with various amounts of PbBr_2_. **Fig. S2.** J–V curves of the control device at different scan directions.

## Data Availability

All the data are fully available without restrictions.

## Abbreviations

3D: Three-dimensional
2D: Two-dimensional
*PCE*: Power conversion efficiency
PVSCs: Perovskite solar cells
PbBr_2_: Lead (II) bromide
PbI_2_: Lead (II) iodide
BAI: N-butylammonium iodide
MAI: Methyl-ammonium iodide
PC_61_BM: Phenyl-C61-butyric acid methyl ester
DMSO: Dimethyl sulfoxide
BCP: Bathocuproine
ITO: Indium tin oxide
*J-V*: Current density-voltage
SEM: Scanning electron microscope
AFM: Atomic force microscopy
RMS: Root-mean-squared roughness
PL: Photoluminescence
*V*_oc_: Circuit voltage
*J*_sc_: Short-circuit current density
*FF*: Fill factor
