# Supplementary material for: Stable High-Efficiency Two-Dimensional Perovskite Solar Cells Via Bromine Incorporation
Source: Nanoscale Res Lett. 2020 Oct 1;15:194. doi: 10.1186/s11671-020-03406-w (PMC7530156; doi:10.1186/s11671-020-03406-w)
Supplement: Supplementary file 1 — Additional file 1: Fig. S1. Statistic distribution for (a) Voc, (b) Jsc, (c) FF, and (d) PCE of 2D PVSCs based on BA2MA4Pb5I16-10xBr10x films with various amounts of PbBr2. Fig. S2. J–V curves of the control device at different scan directions. [file 11671_2020_3406_MOESM1_ESM.docx]

**Supplementary Material**

# Stable high efficiency two-dimensional perovskite solar cells via bromine incorporation

Feng Han^1*^, Wenyao Yang^2^, Hao Li^3^, Lei Zhu^4^

^1^ Xi’an Technological University, Xi' an 710021, People’s Republic of China

^2^ Chongqing Engineering Research Center of New Energy Storage Devices and Applications, Chongqing University of Arts and Sciences, Chongqing, 402160, P. R. China

^3^ State Key Laboratory of Electronic Thin Films and Integrated Devices, and School of Optoelectronic Science and Engineering, University of Electronic Science and Technology of China (UESTC), Chengdu, Sichuan 610054, China.

^4^ Xi 'an Institute of Applied Optics, Xi' an 710100, People’s Republic of China

^*^ Corresponding author: Feng Han, Email address: 27757642@qq.com

**
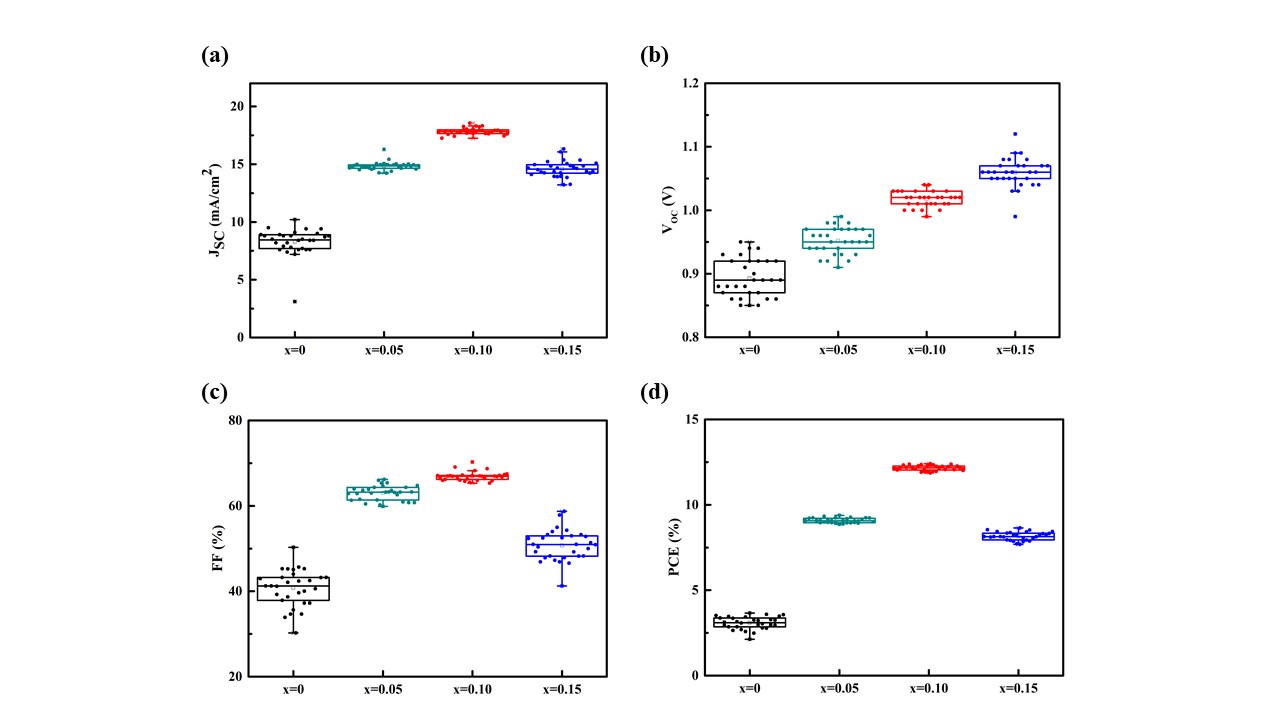
**

**Fig. S1** Statistic distribution for (a) *V_oc_*, (b) *J_sc_*, (c) *FF*, and (d) *PCE* of 2D PVSCs based on BA_2_MA_4_Pb_5_I_16-10_*_x_*Br_10_*_x_* films with various amounts of PbBr_2_.


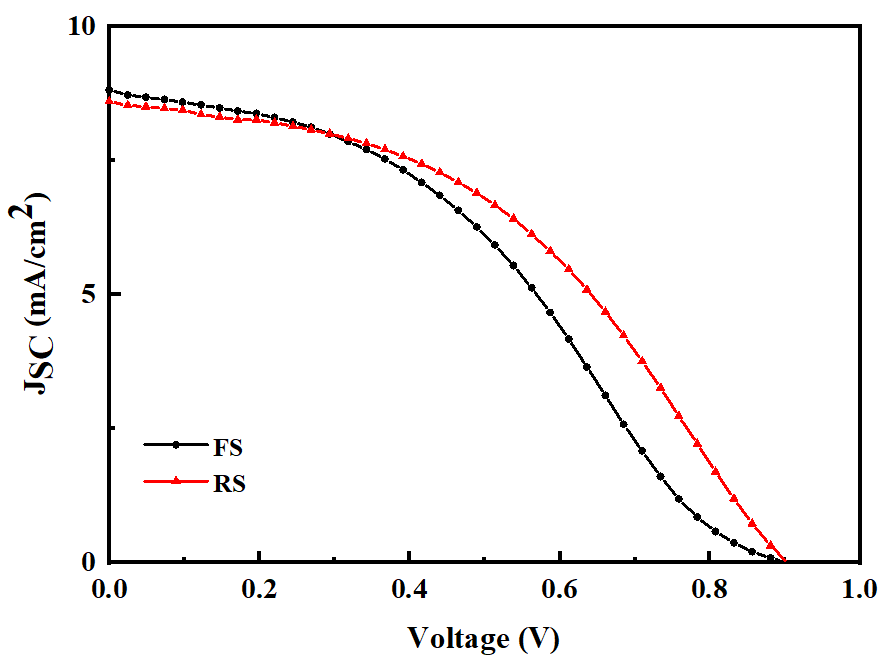


**Fig. S2** J–V curves of the control device at different scan directions.
